# National Scale Operational Mapping of Burnt Areas as a Tool for the Better Understanding of Contemporary Wildfire Patterns and Regimes

**DOI:** 10.3390/s130811146

**Published:** 2013-08-21

**Authors:** Charalampos Kontoes, Iphigenia Keramitsoglou, Ioannis Papoutsis, Nicolas I. Sifakis, Panteleimon Xofis

**Affiliations:** 1 Institute for Astronomy, Astrophysics, Space Applications and Remote Sensing, National Observatory of Athens, Metaxa & Vassileos Pavlou Str, Palea Pendeli, Athens GR 152 36, Greece; E-Mails: ik@noa.gr (I.K.); ipapoutsis@noa.gr (I.P.); sifakis@noa.gr (N.I.S.); 2 Forestry Directorate of Drama, Decentralized Administration of Macedonia and Thrace, Ag. Konstantinou 1, Drama GR 66100, Greece; E-Mail: pxofis@damt.gov.gr

**Keywords:** earth observation, large scale burn area mapping, fire regime Mediterranean ecosystems, wildfire management

## Abstract

This paper presents the results of an operational nationwide burnt area mapping service realized over Greece for the years 2007–2011, through the implementation of the so-called BSM_NOA dedicated method developed at the National Observatory of Athens for post-fire recovery management. The method exploits multispectral satellite imagery, such as Landsat-TM, SPOT, FORMOSAT-2, WorldView and IKONOS. The analysis of fire size distribution reveals that a high number of fire events evolve to large and extremely large wildfires under favorable wildfire conditions, confirming the reported trend of an increasing fire-severity in recent years. Furthermore, under such conditions wildfires affect to a higher degree areas at high altitudes, threatening the existence of ecologically significant ecosystems. Finally, recent socioeconomic changes and land abandonment has resulted in the encroachment of former agricultural areas of limited productivity by shrubs and trees, resulting both in increased fuel availability and continuity, and subsequently increased burnability.

## 1. Introduction

Fire has a long history on Earth which dates back to the first appearance of any terrestrial vegetation, and as a result the coexistence on the planet of the three prerequisites of fire, namely: fuel, oxygen and flame [1]. Ever since, the effect of fire on landscape and ecosystems has been so determinative that the distribution, composition and structure of many biomes across the globe could not be explained by climate and soil alone and fire needs also to be taken into account [1–4], as well as its interaction with climate and soil [5]. In the Mediterranean basin in particular, where the current study was conducted, fire has been one of the main driving forces for the evolution of plants and ecosystems from the onset of Mediterranean climate, determining to a great extent the current structure and composition of Mediterranean landscape [6–10]. The effect of fire on the evolution of plants and ecosystems has been so important that only species possessing adaptive [11] or exaptive [12] traits, allowing them to overcome the detrimental effects of a particular fire regime, could survive in the fire prone areas of the Earth [9,13,14].

Although fire existed well before the appearance of humans on Earth, the interaction between man and fire, and the use of the latter for improving man's living conditions, has significantly altered the fire regime in many areas across the globe, mainly by increasing the frequency of wildfires [1]. The increase of the catastrophic impacts of wildfires coupled with the increased concerns for the conservation of natural resources and the contemporary role of the environment on human life, has led to the adoption of fire suppression strategies in many fire prone regions, from the late 19th century [16].

Despite the recent advances in fire fighting tactics and means, and the increased amount of resources allocated on fire suppression, the efficiency of the adopted strategy has been decreasing over the last four decades with both number of fires and burnt area increasing [17]. In [15] it is reported that a significant increase in both the number of fires and the annual area burned from the late 70s in the province of Valencia of Spain, which was attributed, partly on climatic changes trending towards drier summer conditions and partly to socioeconomic changes, urbanisation and countryside abandonment. The same trend in fire numbers and annual area burned was observed in Greece. Based on data available from the Greek Forest Service (reported in [18]) the average number of fires per year increased from 710.11 in the period 1956–1973 to 978.4 in the period 1974–1987. At the same time the average annual area burned increased from 11,574.17 hectares (ha) to 36,103.06 ha for the two periods, respectively. This trend appears to continue, since the average annual area burnt for the period 1980–2006 is 43,237 ha, according to the annual Forest Fires in Europe 2006 report [19]. It should be noted here that the wildfire country statistics, included in those reports, should be interpreted carefully due to adegree of inaccuracy that they contain. Those data are formulated based on the Fire Event Reports submitted by Fire Brigades and the Forest Service, and the fire size is in most cases was only visually estimated. However, they are still indicative of the fact that wildfires continue to constitute a major threat for the environment and the society, often with significant cost in human lives.

A careful reconsideration of the current wildfire management strategy appears to be necessary in order to reduce the devastating impacts of wildfires in ecosystem's ecological integrity, society and economic activity in the future. The development of a more effective wildfire management strategy requires accurate, spatially explicit data on the annual area burned, which will support a better understanding of the wildfire spatial patterns.

Mapping burnt areas in mountainous regions using conventional surveying methods is a difficult, labor intensive and time consuming task due to landscape complexity, in terms of both topography and land use mosaic. Over the last decades significant advances have been made to map fire extent using Earth Observation (EO) data in Greece exploiting advanced remote sensing methods [20–22].

This paper describes the results of an operational EO-based annual national scale burnt area mapping project over Greece covering from the year 2007 to the year 2011. The identification and recording of the burnt areas was achieved through the implementation of a remote sensing method explicitly developed at the National Observatory of Athens (NOA) for Burn Scar Mapping (BSM, henceforth the BSM_NOA method). The applied BSM_NOA method [22] was developed and deployed in the framework of the GMES European programme, and aims at contributing to a standardised and homogeneous mapping of burnt areas and related vegetation damages in the EU member states. It has already been presented to the Ministry of Environment, Energy & Climate Change, and it has been approved as an accurate method, with a high spatial precision (0.5–1 ha), at desirable mapping scales ranging from 1:10,000 to 1:50,000, and quick production of burnt area maps, which can be as quick as two months after the end of the fire season, greatly supporting the reporting and planning needs of the operational users nationwide.

Fire Size Distribution (FSD) was examined using the obtained burnt area maps. FSD is an important spatial indicator of the fire regime of a region, and it can provide useful information on the efficiency of the applied fire suppression policy [23]. Although the time period from which FSD was calculated is short, the existence within the dataset of a season of extreme wildfire activity and seasons of moderate and low activity can provide useful insight on the efficiency of the currently applied wildfire suppression under different circumstances.

The distribution of burnt areas was analyzed in relation to elevation and land cover type. Recent studies have pointed out a shift in burning patterns over the last century with more fires occurring on higher altitudes, outside the Mediterranean climate distribution zone [24,25], leading to concerns about the future of ecosystems in those elevation zones. In the current study the elevation distribution of the burnt areas was analyzed to find out to what extent this trend occurs, especially under extremely favorable fire conditions.

The distribution of wildfires is strongly affected by the land cover types, with some of them being much more favorable to fire. A regional study on the distribution of wildfires [26], suggested a shift in the burning patterns with some land cover types contributing more than usual to the bunt areas, under extremely favorable fire conditions. This trend is examined in the current study at a national scale and for the current study period.

The aim of the current study was first of all to report on the efficiency of the BSM_NOA method in mapping burnt areas on a national scale. Such a method could support the development of a time series spatial database, which can be analyzed in combination with other spatial datasets, leading to the better understanding of contemporary wildfire spatial patterns. Further, the obtained burnt area maps are analyzed to determine the FSD within the study period, as well as to determine the distribution of wildfires in relation to elevation and land cover type. The results are discussed with emphasis on the recent changes on the fire regime of Greece and the reported climate change trends which are expected to significantly affect the wildfire spatial patterns.

## 2. Material and Methods

### 2.1. BSM_NOA Service Data

Annual national scale burn scar maps were produced from multispectral satellite imagery. The majority of the images were from the Landsat-TM satellite but other sensors of higher spatial resolution, namely SPOT-4, FORMOSAT-2, and WorldView, were additionally used in certain occasions when either a higher level of detail was required by the operational users due to the severity of the event, or simply because suitable cloud-free Landsat images were unavailable. The number of processed images per sensor and year, as well as the geographic regions covered all over Greece by those images, are presented in Table 1.

The image data set required for the BSM_NOA service deployment over Greece, for the 2007 fire season, was provided by the European Space Agency (ESA) in the framework of the RISK-EOS ESA/GSE project (http://www.riskeos.com). The spatial resolution of the Landsat-5 TM and SPOT-4 XS images used in this case is, 30 m and 20 m/10 m (SPOT-4 multispectral bands/SPOT-4 panchromatic band), respectively. Because of the severity of damages in the Peloponnese region in 2007, it was decided to cover the damaged area with the very high spatial resolution images of FORMOSAT-2 (spatial resolution: 2 m at the panchromatic band and 8 m at the multispectral bands). For the same year, the Crete and Aegean Sea Islands regions were not processed as no significant wildfires occurred.

For the 2008 fire season no data were acquired since it was a rather uneventful year with only dispersed and isolated fires that were immediately suppressed. Moreover, this year was the transition from the RISK-EOS ESA/GSE implementation to the SAFER EC/GMES project implementation (http://www.emergencyresponse.eu/), so there was no available funding from the GMES program to acquire satellite data. Conversely, 2009 was a catastrophic year since the northeastern region of the Greek capital, Athens, was severely struck by forest wildfires. The images required for the service were acquired in the framework of SAFER EC/GMES project. With the same dataset it was also possible to identify the fire events that occurred in the 2008 fire season using additionally for verification purposes the fire occurrence reports and fire log files of the National Forest Service and Fire Brigades.

**Figure 1.**
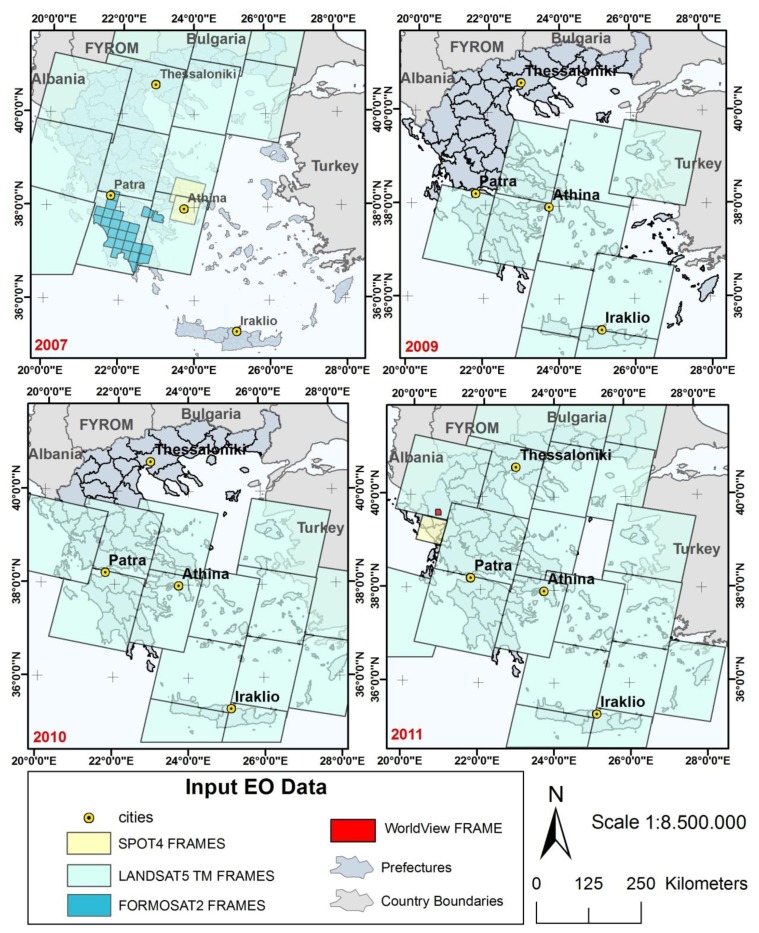
Satellite frames for burnt area mapping in 2007 (**Upper Left**), 2008–2009 (**Upper Right**), 2010 (**Bottom Left**) and 2011 (**Bottom Right**).

Actually for the 2011 fire season 95% of Greece was covered by Landsat-5 TM images and one SPOT-4 image, which covered the Epirus administrative region, except from the peripheral unit of Ioannina for which the very high spatial resolution WorldView imagery (0.46 m at the panchromatic band and 1.84 m at the multispectral band) was used. The satellite frames per year and per sensor used for burn scar mapping, for the fire seasons of 2007, 2008–2009, 2010 and 2011 are shown in Figure 1. It is noteworthy that the satellite coverage of Greece's territory per year was concluded after consultation with the Greek institutional end user involved in the study, the Directorate General for Forests and Natural Environment Protection of the Ministry of Environment and Climate Change, officially nominated as GMES user from Greece in the framework of the SAFER EC/GMES project. This organisation assumes the responsibility to manage forest recovery in the post-fire season at national level. Therefore the decision on the geographic areas to be covered on a seasonal basis, and consequently the satellite data to be acquired, was fully guided by this operational user, based on the reported severity and geographic distribution of the fire events over Greece during the fire season. Consequently, areas that are not covered by satellite data as in are those that according to the end user suggestions did not show any important fire activity to be included in the analysis.

### 2.2. BSM_NOA Methodology Description and Validation

**Figure 2.**
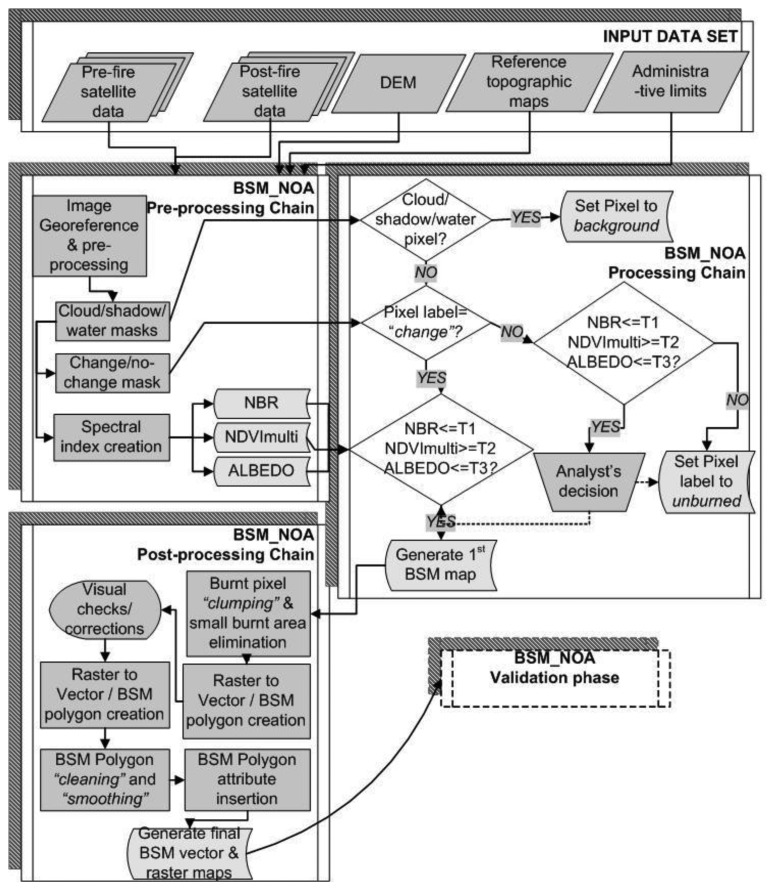
The BSM_NOA processing chain (source: [22]).

The BSM_NOA processing chain is a fixed thresholding method. It relies on the derivation and analysis of uni- and/or multi-temporal spectral indices for biophysical features, such as the Normalised Burn Ratio index, the Albedo index, the Normalised Difference Vegetation Index (NDVI), the multi-date NDVI index, combined with radiometric Change Vector Analysis. The methodological developments together with the validation experiments conducted for assessing the effectiveness and accuracy of the BSM_NOA processing chain are not within the scopes of this article as they have been presented in detail in [22,27]. However, for facilitating the reader to get the full overview of the works realised, the main elements of the BSM_NOA processing chain comprising the three processing phases, known as BSM_NOA Pre-processing, BSM_NOA Core processing, and BSM_NOA Post-processing are briefly outlined hereinafter (Figure 2).

The *Pre-processing phase* comprises the following tasks:

- Satellite data radiometric normalisation to render the multi-date image acquisitions to be of comparable radiometric values, and automatic geo-referencing of the images to the national cartographic projection system (HGRS87) (fully automatic process).
- Cloud, water, and shadow mask generation (fully automatic process).
- Calculation of radiometric change vectors and generation of a change/no-change pixel masks as in [27] (fully automatic process).
- Derivation of uni- and/or multi-temporal spectral indices of physical parameters, namely uni/multi-temporal Normalised Difference Vegetation Index (NDVI), Albedo and Normalised Burn Ration Index (NBR) (fully automatic process). Definition of appropriate index thresholds, through applying a supervised process based on visual inspection of the results over specifically selected test sites. To be noted that the definition of the thresholds varies depending on the sensor and the geographic/landscape characteristics of the area. The spectral indices are calculated as:

The *Normalised Burn Ratio index (NBR)*:

$$\text{NBR}=\frac{\left(R_{\text{NIR}}-R_{\text{MIR}}\right)}{R_{\text{NIR}}+R_{\text{MIR}}}$$

where R*_NIR_* and R*_MIR_* are the radiances in the near infrared and middle infrared parts of the electromagnetic spectrum, respectively.

The uni- and/or multi-temporal *Normalised Difference Vegetation Index (NDVI & NDVI_MULTI_)*:

$$\text{NDVI}=\frac{\left(R_{\text{NIR}}-R_{\text{RED}}\right)}{R_{\text{NIR}}+R_{\text{RED}}}{\text{NDVI}}_{\text{MULTI}}={\text{NDVI}}_{\text{PRE}-\text{FIRE}}-{\text{NDVI}}_{\text{POST}-\text{FIRE}}$$

where R*_RED_*, and R*_NIR_*, denoting the radiances in the red and near infrared parts of the spectrum, and NDVI_MULTI_ denoting the multi-temporal NDVI index.

The *Albedo Index*:

$$\text{ALBEDO}=\frac{R_{\text{NIR}}+R_{\text{RED}}}{2}$$

The *Core Processing phase* applies thresholding of the derived spectral indices in order to separate between the burnt and non-burnt areas (fully automatic process). In order to resolve for classification ambiguities at this processing level, the first burn scar classification results are compared against the derived change/no-change pixel maps, output of the radiometric vector change analysis [27] (hybrid process comprising visual inspection of the results).

In the *Post-Processing phase*, the following refinement tasks are performed:

- Removal of classification errors through application of a median filter to resolve from salt and pepper noise, and then elimination of objects that are smaller in size than the specified minimum mapping unit (MMU) of 1 ha (fully automatic process).
- Generation of GIS compatible burn scar polygons by applying usual raster to vector operations.
- Manual enhancement of the thematic content of the resulted BSM vector layer by, (a) cleaning out obvious errors of unnecessary polygons, and (b) aggregating islands of smaller BSM polygons spread out in the same locality in order to create bigger and more representative to the fire event BSM polygon.
- Generation of the attribute information assigned to the resulted BSM polygons such as, the total size of the burnt area, and the surface of the vegetation types affected according to the CLC data base. Moreover, using existing geo-spatial layers and fire brigade logs, additional attribute information is also added to each polygon such as the administrative related data (municipality name, toponyms, geographic region), and the fire event's ignition and suppression dates.

Given the BSM_NOA processing chain delivers a standardised GMES service, that has been developed in the framework of the RISK-EOS ESA/GSE program, and consolidated and further enhanced in the framework of the SAFER EC/GMES project, it has been repeatedly validated through the years based on a specific validation plan approved by ESA and EC for the GMES purposes. This validation plan clearly identifies the integrity and appropriate accuracy standards to be met by the delivered BSM products. In this framework the accuracy level of the BSM_NOA method was assessed several times in the past using independent *in situ* collected reference data provided by the regional Forestry Services over Greece. In general, the reference datasets were vector or analog maps depicting the damaged areas in the scale of 1:50,000 or better, generated through dedicated *in situ* field campaigns or using large-scale aerial photography of much higher spatial resolution. The detailed presentation of the validation protocol applied in the framework of the RISK-EOS ESA/GSE and SAFER EC/GMES projects is outlined in [22]. Briefly the accuracy figures were expressed in terms of: (a) detected area efficiency, (b) skipped area rate (omission error), and (c) false area rate (commission error). These accuracy figures were calculated on the basis of the following formulae:

$$\begin{array}{l} \text{Detected Area Efficiency}=\frac{\text{DBA}}{\text{DBA}+\text{SBA}}\\ \text{Commission Error}(\text{Flase Area Rate})=\frac{\text{FBA}}{\text{DBA}+\text{FBA}}\\ \text{Ommission Error}(\text{Skipped Area Rate})=\frac{\text{SBA}}{\text{DBA}+\text{FBA}} \end{array}$$

where DBA is the Detected Burnt Area (common area between the generated burn scar polygon and the reference *in-situ* polygon), FBA is the False Burnt Area (area included in the generated burn scar polygon but not in the reference in-situ polygon), and SBA is the Skipped Burnt Area (area included in the reference *in-situ* polygon but not in the generated burn scar polygon).

In general, as first calculated and presented in the official reports of the RISK-EOS ESA/GSE project, but also calculated and published in [22], using the aforementioned validation protocol in the Mani (23 August 2006), and the Parnitha (19 July 2007) fire events that were very big in size (∼6,000 ha and 4,500 ha, respectively), and highly complex in terms of landscape morphology and land cover types affected, the overall efficiency of the BSM_NOA method for burnt area detection were estimated to be as high as 88%, with the omission errors (‘skipped areas’) ranging between 13%–23%, and the commission errors (false alarms) to be as low as 3%–6%. Moreover, as reported in the RISK-EOS ESA/GSE project deliverables, all of the fire events documented in the fire logs greater than 0.5–1 ha, had been identified and mapped by the BSM_NOA method using high resolution satellite data (Landsat TM, SPOT XS). Also as noted in [22] the rather higher omission errors reported, affecting the overall efficiency level as well, did not correspond to real cases of skipped burnt areas. In reality, they corresponded to ‘island areas’, not affected by the fire, located inside greater burned area polygons, the so-called ‘donut’ polygons. Therefore these polygons were correctly annotated by the BSM_NOA processing chain to non-burnt surfaces, however, the fact that they were not included in the reference datasets, they have been erroneously returned as skipped areas.

Moreover, in the framework of the SAFER EC/GMES project, the BSM_NOA method has been validated by independent evaluators for the needs of the European Commission in a geographic context that excluded Greece. The method's accuracy was assessed over the complex forested landscape of the island of Corsica. In this context, the method was compared in terms of the thematic quality and several other criteria focusing on the assessment of the operational maturity and accuracy standards required by the GMES stakeholders, with other similar methods also used in the framework of GMES for Burn Scar Mapping. The results of this external validation exercise are explicitly stated in [28], delivered in the framework of the SAFER EC/GMES project.

The test case was chosen thanks to the ground truth provided by Office National des Forêts of Corsica (ONF). Two fires which occurred in August 2003 in the communes of Tolla and Aullène in the South of Corsica were considered. ONF provided the ground truth, in terms of fires perimeters. Following this test scenario, the NOA BSM service was qualified -top of its class- as an end-to-end service for fire related Emergency Support activities for integration to operational scenarios all over Europe. The thematic accuracy was originally checked considering the shapefile provided and comparing it to the ONF perimeters. Then all map elements were considered, to confirm the reliability of the information content, the consistency of the information support and the usability of the product.

JRC performed the scientific validation of the NOA BSM product using ground truth fire perimeters and concluded to high producer's and user's accuracies (above 85%) and low commission and omission errors (below 15%). The detailed validation results for the two test areas in Corsica are depicted in Table 2.

### 2.3. Analysis of Burnt Patterns

Fire size distribution was the first analysis performed. The identified fires were classified in five size classes, namely: <10, 10–100, 100–1,000, 1,000–5,000 and >5,000 ha, in order to identify the percentage of large fires in relation to the total number of events. The results obtained were used to assess the effectiveness of the applied wildfire management strategy in relation to the risk imposed by wildfires, given the contemporary landscape and climatic patterns.

The obtained GIS layers containing the fire polygons where further used in combination with other spatial data sets in order to identify the burnt patterns during the studied time period. For the identification of the altitudinal distribution of fires the ASTER Digital Elevation Model was employed and used to divide Greece into four altitudinal zones: namely: 0–500, 500–1,000, 1,000–1,500 and >1,500 m above the sea level (a.s.l), using the software eCognition Developer 8.7. The percentage of burnt areas falling within these altitudinal zones was then calculated.

The main land cover types present within the most fire prone altitudinal zones were identified using the CORINE Land Cover 2000 classes of Level 3 (http://www.eea.europa.eu). The analysis was restricted to natural and semi-natural land cover types that participated at least by 1% in the total burnt area in at least one fire season.

The susceptibility of each of those land cover types to fire was assessed using Chi-square analysis. The area burnt in hectares of each of the analyzed land-cover types, each year, was used as the observed frequency while the area in hectares covered by each land cover type respectively, in the entire country adjusted for the total area burnt for each particular year, was used as the expected frequencies. The “null” hypothesis is that if all land cover types respond in the same way to wild fires, then one would expect the relevant contribution of each of them to be proportional to its contribution to the total land cover. The analysis was done using the software Statistica 8 [29].

## 3. Results

**Figure 3.**
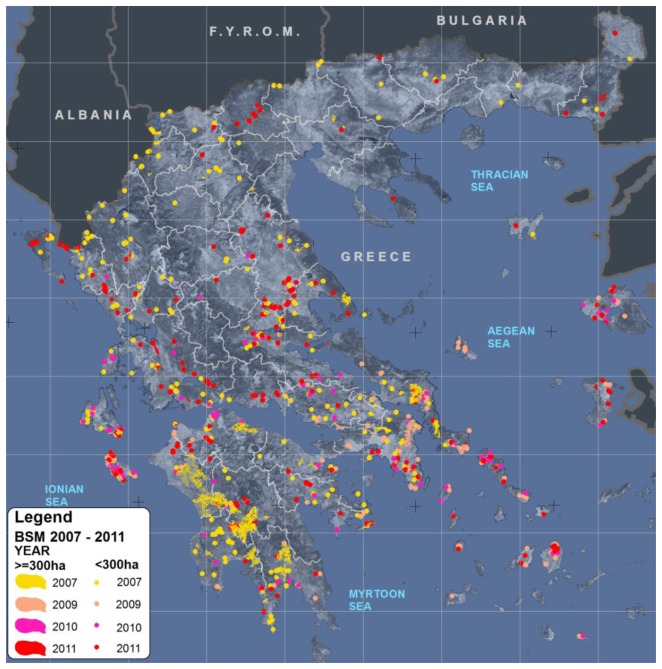
Mapping of burnt areas for the service years 2007–2011. Colored polygons corresponding to burnt areas ≥300 ha, and dots to burnt areas <300 ha, indicate the locations and therefore the geographic distribution of the fire events.

The wildfires mapped were those with fire size greater than 1 ha. Figure 3 shows the distribution of burnt areas at national scale, as mapped from the BSM_NOA service, for the fire events that occurred in Greece from year 2007 to 2011. It is clear that most fire events occurred in central and Southern regions of Greece, while the Peloponnesus was the most severely damaged region, followed by SE Attica and Euboea.

The results of the BSM_NOA service deployment, for years 2007, 2008, 2009, 2010 and 2011, are synoptically presented in, depicting the total number of fire events and the associated burnt areas. As no satellite imagery was available for the year 2008, mapping of the 2008 fire events was based on images of the year 2009 and the two fire seasons are treated as one in both mapping and the analysis of the resulted dataset.

The year 2007 was by far the most devastating year (Table 3 and Figure 3), inflicting great damages to southern Greece. A total of 256 events were detected leading to a total of 195,018 ha of burnt areas. In 2008 and 2009 the total burnt area and the number of fires were limited compared to 2007, but one major catastrophic event struck near the capital city of Athens in central Greece. A total of 13,045 ha were burnt in Attica, whereas 5,395 ha were burnt near Karystos in Euboea. On the other hand 2010 was a year with exceptionally low total burnt area and a small number of fire events. The characteristics of 2011 were the large number of fire events, which was not accompanied by a large size of total area burnt. In 2011 there was only one major incident in north-east Greece with significant, however, ecological consequences as discussed below.

**Figure 4.**
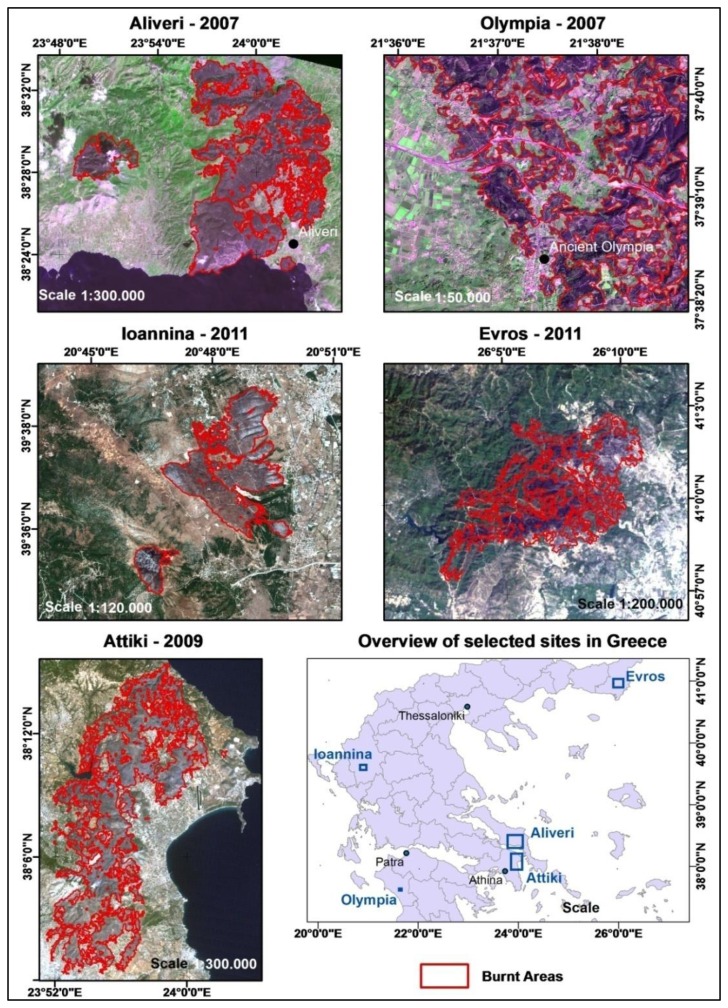
Burn Scar Maps (BSM) for the fire events at (**Upper Left**): Aliveri (SPOT-4 image, spatial resolution of 20 m); (**Upper Right**): Olympia archaeological site (FORMOSAT-2 image, spatial resolution 2 m); (**Middle Left**): periphery of Ioannina (WorldView-2 images, spatial resolution of 1.84 m); (**Middle Right**): Evros area (Landsat-5 TM, spatial resolution of 30 m) and (**Bottom Left**): NE Attica (Landsat-5 TM, spatial resolution of 30 m).

Whilst Figure 3 illustrates the national scale service, it is of interest to present the level of detail of the service at local scale. As the detail level depends on the source satellite images used, five maps are presented at different scales (from 1:20,000 to 1:300,000) for the reader to appreciate the accuracy of the proposed approach. Examples of five different burn scar maps are presented in Figure 4, each of which corresponds to a source satellite image of different spatial resolution.

**Figure 5.**
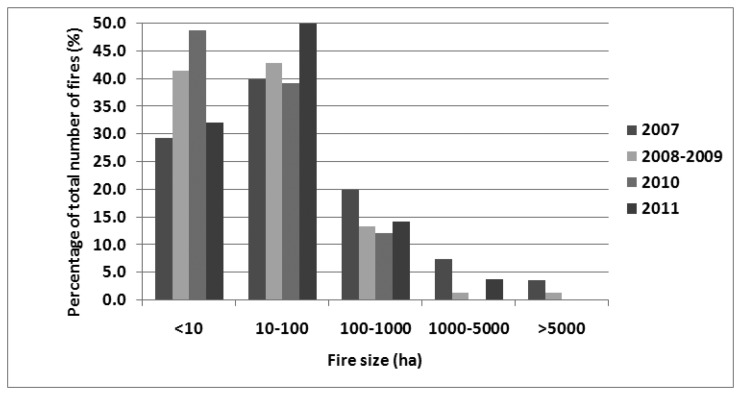
Fire size distribution for the studied fire seasons 2007–2011.

The fire size distribution (Figure 5) shows that in 2007 approximately 11% of the fires had a size of more than 1,000 ha, while nine fires (3.5%) burnt more than 5,000 ha each. In 2008–2009 2.5% of fires burnt more than 1,000 ha and two fires (1.2%) burnt more than 5,000 ha. The nine largest fires in 2007 burnt 142,716.5 ha or 73.2% of the total, and in 2008–2009 the two largest fires burnt 18,441.2 ha or 57.3% of the total. If those mega fires had been successfully suppressed then the total burnt area would be approximately 52,302 ha in 2007 and 13,735 ha in 2008–2009, slightly above and well below the decade's average respectively. In 2010 and 2011 the large majority of wildfires burnt less than 100 ha each, and in none of these years did a single event burn more than 5,000 ha. However, in 2011, which was a year where the burnt area did not exceed the annual average for the period 1980–2005, 3.7% of the events resulted in fires with an area burnt exceeding 1,000 ha, and one of them resulted to the severe destruction of the very important raptors habitat of Dadia National Forest in the north of Greece. The relatively smaller number of fires sized lower than 10 hectares compared to the fires with size of 10–100 ha, is an observation that deviates from the assumed power-law distribution of fire size distribution. However, this is not an actual pattern bur rather due to the minimum mapping unit which was set to 1 ha, omitting many very small fires.

**Figure 6.**
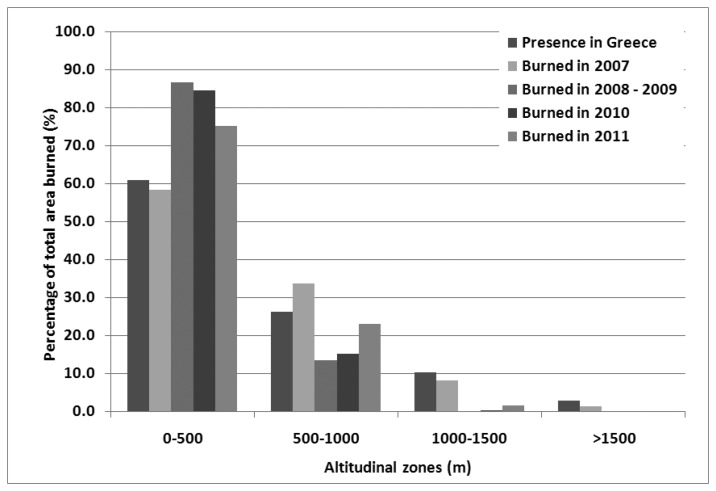
Altitudinal distribution of fires within each of the distinguished altitudinal classes.

Concerning the altitudinal distribution of fires, Figure 6 indicates that the great majority of burnt area falls within the 0–500 m elevation zone (61%–86.5%), followed by the 500–1,000 m elevation zone (13.4%–33.6%). The year 2007 was the only case where a significant proportion of the burnt area is found in altitudes above 1,000 m (9.3%), while for the rest of the years this proportion varied from 0.1% to 1.6%. The following analysis on the contribution of each land-cover type in the burnt area is restricted to the area below 1,000 m which constitutes the typical fire occurrence altitudinal zone.

**Figure 7.**
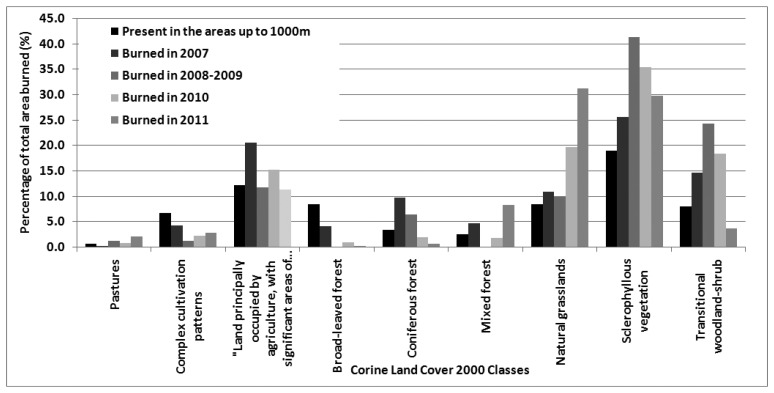
Contribution of each CLC 2000 level 3 classes on the total area burnt per year.

Figure 7 shows the relative contribution of each land cover class to the total area burnt per year, while its presence in the entire country is also shown as a reference. Those results were further analyzed using chi-square, separately for each year and the results are shown in Table 4.

Sclerophylous vegetation and natural grasslands are the only land-cover types with observed frequencies higher than expected in all study years indicating that these land cover types form the main fuel sources. Broad-leaved woodland and complex cultivation patterns, on the other hand, have observed frequencies lower than the expected in all years indicating the resistance of those land-cover types to fire. Of significant importance is the variation observed in the contribution on the total burnt area of the class ‘Land principally occupied by agriculture with significant areas of natural vegetation’. While in most years its contribution to the total area burnt is proportional to its contribution to the total land cover, in the extreme year of 2007 it contributes to the total area burnt almost twice as much as its contribution to the total land cover. Its increased contribution which is observed also in 2010 is of minor importance given the extremely small area burnt that year.

## 4. Discussion

The application of the BSM_NOA methodology over Greece has provided the scientific community, forest services, ecologists, and landscape managers, with objective and accurate yearly assessments of wildfire affected areas. The reported results provide insights on method's flexibility, timeliness, and efficiency especially when applied to very large volumes of multi-sensor high and very high spatial resolution satellite data such as LANDSAT TM, SPOT, WorldView, FORMOSAT 2, *etc*. Moreover, the burnt area maps generated here when they are used in combination with fuel maps of high accuracy [30] can provide extremely useful insights for the effective planning of fire prevention and allocation of resources for fire suppression.

The southern and more arid parts of Greece experience a higher number of wildfires than the northern and wetter parts, revealing a climatic gradient that strongly affects fire regime, as pointed out in other studies in Mediterranean Europe [15,31], and other areas with Mediterranean type climate [5]. The high number of wildfires in Central Greece and Attica in particular, could also be attributed to the extremely high population density in this area, where almost half of the population of Greece is concentrated, resulting in an increased number of ignition sources [32]. Although [33] revealed a negative correlation between population density and potential for a large fire, the experience of 2009 fires in Attica as well as those of years previous to 2007 suggest that Attica is a fire hot-spot, which might also be related to the conflicts over land ownership in Greece, as a result of the absence of a comprehensive cadastral which could resolve those conflicts.

The fire size distribution revealed that the large majority of fire events burn relatively smaller areas, which is in accordance to the typical power-law FSD. However, in 2007 11% of the events resulted in large or extremely large fires, which are the ones that determine the post-fire landscape patterns and cause the most detrimental effects to environment and society [34]. The fire season of 2007 was the worst ever recorded in terms of area burnt, although the number of fires was almost equal to the one in 2011, and according to the Forest Fires in Europe 2007 [35] report it was high but not the highest since 1980. The entire year of 2007 and the summer period in particular was characterized by extreme weather conditions with the highest temperatures recorded for almost a century, three consecutive heat waves from June to August and wind patterns that favoured the spread and intensity of fires [36,37]. If these extreme weather conditions were an isolated incident with very low probability to happen again in the future, then the problem of wildfire management could be perhaps resolved with some minor alterations to the one currently applied, but this is far from what it is expected. As stated in [36,37], 2007 represents an example of how the weather condition will be like by the end of the century as a result of climate change. Further, the IPCC Forth Assessment Report [38] suggests that summer temperatures and precipitation are likely to increase and decrease, respectively, during the following decades leading to weather patterns that we have possibly never experienced or recorded before. Similar projections are given by other studies as well, including [15,39–41]. If one takes also into account the already reported trend of increasing burnt area and number of fires over the last decades, one could conclude that the currently applied wild fire management strategy, which allows more than 10% of fire incidents to evolve into large or extremely large fires, under weather conditions which are likely to occur often in the future, can no longer be considered adequate.

According to [25] the percentage of burnt area at high altitudes where upland conifers prevail has increased from 1.7% in the period 1960–1983 to 3.7% in the period 1983–2004, suggesting an expansion of wildfires into higher altitudes, probably as a result of climatic changes [24,25]. The current study reveals that although the great majority of fires burn areas bellow 1000 m, under extremely favorable fire conditions, such as in 2007, 9.3% of the burnt area occurs at altitudes above that limit, confirming the reported trend. This observation is of particular importance given the significant risk imposed for the upland coniferous forest communities by this shift in the burning patterns. Those communities include unique habitat types such as the *Abiescephalonica* forests of Attica and Peloponnese. Such habitat types, with high conservation value, do not possess any trait to overcome the detrimental effects of fire due to the fact that their distribution zone falls above the typical fire prone zones of Greece [26]. Thus, if in such areas wildfires become a periodic and not just a random effect, as a result of the foreseen climatic changes, then the long term, existence of those communities is under serious threat [24].

Regarding the response of the various land-cover types to wildfires the current study suggests that sclerophyllous vegetation formations constitute the main source of fuel in most study years and only in 2011 natural grasslands contributes to a slightly higher amount. Sclerophyllous vegetation formations consist of species such as *Quercuscoccifera*, *Arbutus unedo*, *Phillyrealatifolia*, *Q. Ilex*, *Cistus sp.*, *Calicotomevilosa* and others, which all possess traits that allow them to overcome the detrimental effects of fire. These include resprouting from below ground organs, such as roots or lignotubers [9,42,43] or germination from dormant seeds stored in the underground seedbank, after the dormancy is released by the action of a fire related germination cue [9,44,45]. Under the extreme conditions of 2007, however, the land-cover type “Land principally occupied by agriculture with significant areas of natural vegetation” seems to significantly contribute to the burnt area while for the rest of the years it also contributes at a rate proportional to its contribution to the total land cover. According to [26], who observed the same phenomenon in the wild fires of Peloponnese, this land-cover type represents areas which were traditionally used for agriculture, but due to the recent socioeconomic changes they have been abandoned to a large extend and have been encroached by shrubs or trees, leading to increased fuel availability and continuity. This trend of land abandonment and expansion of Mediterranean shrublands has also been observed by other studies [15,44,46], which point out the significant role of these landscape changes in the transition from a fire regime with frequent small fires to a fire regime with less frequent stand replacing fires. Fire suppression, as it is currently applied, has also been considered by many studies responsible for this transition in the fire regime [23,47–49]. Long term fire suppression results in increased fuel availability and continuity, which in turn results in large and intensive wildfires that cannot be effectively controlled and suppressed. Although these findings have been disputed by other authors as in [50] it is certainly true for some areas.

## 5. Conclusions

The present study provides concrete evidence that the BSM_NOA remote sensing mapping method offers advanced capabilities for burnt area mapping, as far as the criteria for thematic accuracy, timeliness, and large geographic coverage extent are concerned. In general, the EO based approaches are by far exceeding the mapping standards established by foresters at any administrative level (region/country/continent) for supporting actions relating to wildfire recovery management and emergency support and response. The specific BSM_NOA method has been characterized by high flexibility and transferability to any geographic area over Greece, accommodating an interactive approach for the definition and fine tuning of the spectral thresholds for burn scar delineation depending on the type, and the spectral/spatial resolution of the satellite data used. On the basis of these remarks, the use of satellite data in dedicated processing chains as the BSM_NOA, form suitable and robust solutions for operational burn scar mapping at European or national level.

The analysis of burnt areas in relation to their size, elevation and land cover distribution suggests that significant changes on the fire regime are already occurring, and will most likely continue to occur as has already been suggested in several studies. More large and catastrophic fires may be expected in the future if those trends continue and the projected climatic changes confirmed. Moreover, wildfires seem to be expanding both their land cover preferences, as well as their altitudinal distribution, thus threatening ecologically significant habitats. Consequently, the currently applied wild fire suppression policy appears to be rather ineffective in protecting natural ecosystems, properties and human life under the foreseen climatic and socioeconomic changes. A new approach is needed which will be based on an in depth understanding of the contemporary wildfire spatial pattern. One potential field of investigation in the Mediterranean region is the use of fuel management methods, such as prescribed burning, which could possibly reduce the potential of a fire event to evolve into a large or extremely large wildfire.

## Figures and Tables

**Table 1.** Satellite imagery used for the annual national scale burnt-area mapping (BSM_NOA service delivery) for the years 2007–2011.

**BSM_NOA Service Year**	**Landsat-5 TM Images**	**Additional Images**	Administrative Regions Covered*
2007	15	2 × SPOT-4, 72 × FORMOSAT-2	Peloponnesus, Central Greece, Ionian Sea Islands, Epirus, Thessaly, Macedonia, and Thrace
		
2008–2009	10	-	Peloponnesus, Attika, Central Greece, Crete, Central Aegean Islands and Cyclades.
		
2010	14	-	Peloponnesus, Attika, Central Greece, Ionian Sea Islands, Thessaly, Crete, Central and South Aegean Islands.
		
2011	20	1 × SPOT-4, 1 × WorldView2	Peloponnesus, Attika, Central Greece, Ionian Sea Islands, Thessaly, Crete, Thrace, Aegean Islands, Macedonia and almost the entire Epirus region.

* Administrative regions at NUTS II level.

**Table 2.** External thematic accuracy assessment of the BSM_NOA chain.

**Region**	**Tolla**	**Aullène**
Commission error	13.10%	5.76%
Omission error	9.32%	12.70%
Producer's accuracy	90.68%	87.30%
User's accuracy	86.90%	94.24%
Fuzzy Kappa	0.843	0.892

**Table 3.** Number of fires and burnt area in total, and separately for the Northern and Southern regions of Greece.

**Year**	**Total**		**South**		**North**	
		
	**Number of Fires**	**Burned Area (Ha)**	**Number of Fires**	**Burned Area (Ha)**	**Number of Fires**	**Burned Area (Ha)**
**2007**	256	195018	174	180727	82	14291
**2008–2009**	159	32175	159	32175	-	-
**2010**	125	6401	123	6381	2	20
**2011**	246	30711	197	22891	49	7820

**Table 4.** Chi-square results for each study year and landcover type. Positive values in the Observed-Expected column indicates fire proneness.

**Corine 2000 Land Cover Type Level 3**	**2007** **X^2^= 40,395.48, *p* < 0.001**		**2008–2009** **X^2^ = 21,195.68, *p* < 0.001**		**2010** **X^2^= 2,726.47 *p* < 0.001**		**2011** **X^2^= 25,961.49 *p* < 0.001**	
			
	$\frac{\text{Obs}-\text{Exp}}{\text{Exp}}$	**Chi Square Contribution**	$\frac{\text{Obs}-\text{Exp}}{\text{Exp}}$	**Chi Square Contribution**	$\frac{\text{Obs}-\text{Exp}}{\text{Exp}}$	**Chi Square Contribution**	$\frac{\text{Obs}-\text{Exp}}{\text{Exp}}$	**Chi Square Contribution**
Pastures	−0.8396	910.53	0.8177	141.27	0.0913	0.356	1.8843	716.95

Complex cultivation patterns	−0.4399	2,814.83	−0.8374	1,668.62	−0.7041	238.409	−0.6304	903.69

Land principally occupied by agriculture, with significant areas of natural vegetation	0.5097	6,898.76	−0.1276	70.67	0.1045	9.589	−0.1786	132.38

Broad-leaved forest	−0.5803	6,187.08	−0.9943	2,970.68	−0.9069	499.507	−0.9898	2,813.80

Coniferous forest	1.6033	18,657.87	0.7401	650.29	−0.5005	60.099	−0.8565	832.20

Mixed forest	0.6525	2,305.77	−0.9840	857.67	−0.3612	23.362	1.9265	3,142.02

Natural grasslands	0.1560	445.59	0.0532	8.48	1.0780	702.983	2.3189	15,383.17

Sclerophyllous vegetation	0.2043	1,728.73	0.9650	6,310.88	0.6536	585.098	0.3959	1,015.10

Transitional woodland-shrub	0.6176	6,704.81	1.7212	8,517.13	1.0222	607.071	−0.6099	1,022.19
